# Epigenetic regulation and therapeutic strategies in ulcerative colitis

**DOI:** 10.3389/fgene.2023.1302886

**Published:** 2023-12-15

**Authors:** Liwei Yan, Chao Gu, Shanyu Gao, Benzheng Wei

**Affiliations:** ^1^ The First Clinical College, Shandong University of Traditional Chinese Medicine, Jinan, China; ^2^ Departments of Anorectal Surgery, Affiliated Hospital of Shandong University of Traditional Chinese Medicine, Jinan, China; ^3^ Center for Medical Artificial Intelligence, Shandong University of Traditional Chinese Medicine, Jinan, China; ^4^ Qingdao Academy of Chinese Medical Sciences, Shandong University of Traditional Chinese Medicine, Jinan, China

**Keywords:** epigenetics, DNA methylation, histone modification, non-coding RNAs, ulcerative colitis

## Abstract

Ulcerative colitis (UC) is an inflammatory bowel disease, and is characterized by the diffuse inflammation and ulceration in the colon and rectum mucosa, even extending to the caecum. Epigenetic modifications, including DNA methylations, histone modifications and non-coding RNAs, are implicated in the differentiation, maturation, and functional modulation of multiple immune and non-immune cell types, and are influenced and altered in various chronic inflammatory diseases, including UC. Here we review the relevant studies revealing the differential epigenetic features in UC, and summarize the current knowledge about the immunopathogenesis of UC through epigenetic regulation and inflammatory signaling networks, regarding DNA methylation, histone modification, miRNAs and lncRNAs. We also discuss the epigenetic-associated therapeutic strategies for the alleviation and treatment of UC, which will provide insights to intervene in the immunopathological process of UC in view of epigenetic regulation.

## Background

Ulcerative colitis (UC) and Crohn’s disease (CD) are the major forms of inflammatory bowel disease (IBD). UC is considered as an immune-related inflammatory disease with a noticeable prevalence in developed countries (>0.3%) (Mulder et al., 2014; Ng et al., 2017). UC is mainly characterized by the lesion at the mucosa and submucosa of the colon and rectum, and patients subjected to UC have a higher risk of developing into colorectal cancer (CRC) owing to the long-time chronic inflammation (Ungaro et al., 2017; Stellingwerf et al., 2019).

Many studies about *in vitro* and *in vivo* experiments and also clinical data have contributed to a better knowledge of the mechanisms of UC. UC is majorly considered to be driven by the collaboration of the genetic, environmental and dysfunctional immune-mediated factors, as well as the disorder of host-microbiota homoeostasis (Porter et al., 2020). Accordingly, the incidence rate of UC is closely related to socioeconomic situation, which is potentially attributed to the radical change of environmental factors and lifestyle (Ruiz-Casas et al., 2021). In recent years, numerous studies based on multi-omics analyses have highlighted the relation between microbial dysbiosis and the status of UC, and identified potential targets, such as *Bacteroides vulgatus* proteases, for treating UC (Schirmer et al., 2018; Shen et al., 2018; Lloyd-Price et al., 2019; Mills et al., 2022). An endoscopic study demonstrated that intestinal bacterial biofilms are present in about 57% of patients with irritable bowel syndrome and 34% of patients with UC, underlying an endoscopic feature with abnormal bile acid metabolism and bacterial dysbiosis (Baumgartner et al., 2021). Therefore, many researches uphold the apply of antibiotics and relevant interventions to treat and prevent IBD, such as the application of prebiotics, probiotics, antibiotics, gene manipulation and fecal microbiota transplantation approaches, which has been previously reviewed (Glassner et al., 2020).

The influences of immune cells, cytokines and related inflammatory signaling pathways have been also reported in the pathophysiology of UC. For example, neutrophils have been reported to facilitate the inflammatory cell death, thus potentiating and amplifying the pro-inflammatory environment in UC (Angelidou et al., 2018; Dinallo et al., 2019). The pro-inflammatory cytokines produced by neutrophils, monocytes and macrophages, including IL-6, IL-1β, and TNF-α, together create an inflammatory milieu and probably promote a pathologic adaptive immune response in UC (Friedrich et al., 2019; Na et al., 2019). Besides, Th2 and Th17 responses play essential roles in the immunopathology of UC, mediated by IL-4, IL-5, IL-13 and IL-23 (Fuss et al., 2004; Kobayashi et al., 2008). Innate lymphoid cells (ILCs) have also been reported to mediate the inflammatory responses mediated by IL-23 in UC (Hepworth et al., 2013). Another study has analyzed the circulating T cells that were isolated from patients with UC and CD, and identified that transcriptional signatures of CD8^+^ T cells about the signaling triggered by IL-7 and TCR ligation are related to the aggressiveness of disease course (Lee et al., 2011). Apart from those immune cell types and cytokines mentioned above, multiple cytokines secreted by antigen presenting cells, effector and regulatory T cells, intestinal epithelial cells (IECs) would act as promising targets for the treatment of UC, including anti-TNF and anti-IL-12 therapies, as well as tofacitinib as cytokine signaling blockers. The relevant studies have been reviewed previously (Neurath, 2014).

Of late, data about the epigenetic, transcriptomic, post-transcriptomic, proteomic and metabolomic modifications in IBD have been accumulated, which contribute to illustrating the complexity of IBD pathogenesis and immunopathology, and also identifying more therapeutic targets for interventions and treatments (Norouzinia et al., 2017). Among these, epigenetic mechanisms that modulate gene expression via different kinds of modifications of DNA, histones and chromatin, have been unraveled to play significant roles in the development of diverse inflammatory diseases including UC (Toyota et al., 2002; Koch et al., 2013; Ballestar and Li, 2017). In this review, we fully summarized currently available studies with regards to the mechanisms of the epigenetic regulation in the immunopathology of UC. We also discuss the epigenetic-associated therapeutic strategies for the alleviation and treatment of UC, which will provide insights to intervene in the pathological process of UC related to epigenetic mechanisms.

## Epigenetic modifications

### DNA methylation

DNA methylation is a basic mechanism of long-term epigenetic modulations to regulate the expression of genes. Its dysregulation leads to abnormal cell function and phenotypes, and in turn, results in the development of complex diseases.

Epigenome-wide association study (EWAS) is a method to detect genome-wide epigenetic variants (especially DNA methylation at CpGs), so that to find the differences statistically associated with phenotypes of interest (Campagna et al., 2021). Novel candidate risk loci have been discovered through three-layer EWAS using intestinal biopsies of UC, some of which were functionally involved in the inflammatory responses, including the serine protease inhibitor *SPINK4*, the complement factor CFI, and the adhesion molecule *THY1* (Hasler et al., 2012).

Genome-wide DNA bisulfite sequencing of mucosal biopsies from treatment-naïve patients with UC and healthy counterparts is used to evaluate the relation between DNA methylation patterns and gene expression levels in UC. Accordingly, hyper-methylation is observed in the genes related to homeostasis and defense, while hypo-methylation in the genes involved in immune responses, such as chemokines and interleukins (Taman et al., 2018). Another study has isolated IECs of the whole colonic biopsies from UC patients, and generated gene expression and genome-wide DNA methylation signatures of the inflamed and matched non-inflamed regions in the colon. Identification of differential methylation patterns through integrative analysis revealed four key genes that inversely correlated between gene expression and methylation state: *ROR1*, *RARB*, *GXYLT2*, and *FOXA2* (Barnicle et al., 2017), emphasizing the importance of colonic IECs and these four genes in the pathogenesis of UC as potential therapeutic targets. Furthermore, the abnormal methylation of tubulin protein TUBB6 was identified as a marker of the progression of UC to invasive diseases, via a whole epigenome analysis of samples of normal, disease-related and dysplasia/neoplasia mucosa in UC patients (Beggs et al., 2018). The methylation level of *ESR1*, *TUSC3* and *PAR2* genes were significantly higher in patients with UC (Arasaradnam et al., 2010; Gould et al., 2016). These previous data suggest that the changes in DNA methylation and their influence on the transcriptome might represent the mechanisms of the immunopathology of UC, less depending on genetic variation. As to the methylation features of the blood, a relevant study has found that *CXCL5*, *CXCL14*, *IL4R*, *IL17C* and *GATA3* were markedly hypermethylated in the peripheral blood of UC patients compared to those of healthy counterparts (Karatzas et al., 2014).

The chronic inflammatory state exposes patients with UC to dangerous signals causing potential pathogenicity and tumorigenicity, and thus UC elevates the risk to develop CRC (Eaden et al., 2001). DNA methyltransferase (DNMT) is elevated in non-neoplastic mucosa and serves as an early event in UC-CRC (Foran et al., 2010; Fujii et al., 2010), implying the crucial roles of DNA methylation in UC-related tumorigenicity. A previous study detected the methylation level of multiple genes in the mucosa from UC-CRC tumors and non-neoplastic colons, and identified the methylated promoters of *MINT1*, *RUNX3* and *COX2* as potential signatures of the occurrence of CRC in UC patients (Garrity-Park et al., 2010). Moreover, the overexpression of death-associated protein kinase (DAPK), and its inactivation mediated by the hypermethylation of *DAPK* promoter region result in the accumulation of genomically-damaged epithelial cells in the inflamed colonic epithelium in UC patients, which may initiate the development of UC-related carcinoma (Kuester et al., 2010).

**FIGURE 1:**
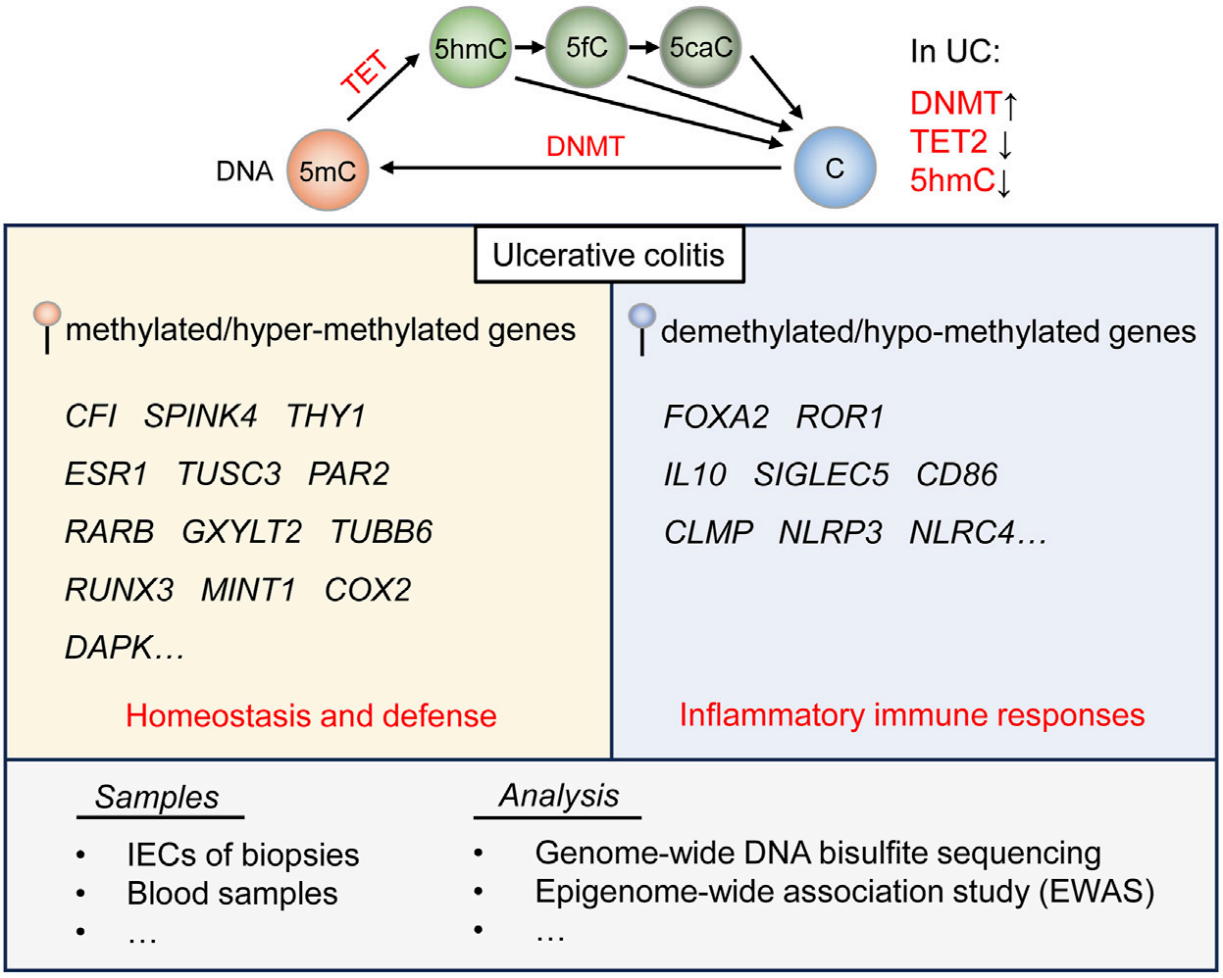
DNA methylation and demethylation regulation in UC. DNA methylation is mediated by DNMT, and demethylation is catalyzed by TET enzymes. DNMT is generally upregulated, while the expression of TET2 and the level of 5 hmC are downregulated in UC. According to diverse clinical samples and analytic tools, genes related to homeostasis and defense are found featured by DNA methylation, and genes involved in inflammatory immune responses by DNA demethylation in UC.

As was stated above, DNA hyper-methylation is proved to play essential roles in the immune dysregulation related with UC. Nevertheless, the function of DNA hypo-methylation in UC is generally overlooked. A relevant study has performed whole transcriptome RNA-sequencing and genome-wide DNA bisulfite-sequencing of mucosal biopsies from patients with severe and mild UC, and found that DNA hypo-methylation of the anti-inflammatory genes is observable in severe UC, including *IL10*, *SIGLEC5*, *CD86*, *CLMP*, *NLRP3* and *NLRC4* (Taman et al., 2021). DNA hypo-methylation of gene *Zbtb7b*, referred to as the Th-inducing POZ-Kruppel factor (Th-POK), could activate the maturation of CD4^+^ T cells and repress the differentiation of double-positive T cells, leading to the secretion of inflammatory cytokines and thus causing colonic inflammation in UC (Xu et al., 2022a). Moreover, DNA methylation is counteracted by the demethylation process catalyzed by TET enzymes, which mediate a series of oxidation reactions and convert methylated 5-methylcytosine (5mC) to 5-hydroxymethylcytosine (5hmC), 5-formylcytosine (5fC) and sequentially 5-carboxylcytosine (5caC) (Wu and Zhang, 2017). A recent study has analyzed colon tissue samples from patients with UC and CRC as well as healthy counterparts, and corroborated that the expression of TET2 and 5hmC levels decrease in those samples from patients with UC or CRC (El-Harakeh et al., 2022) (Figure 1).

### Histone modification

Histone modifications, such as histone acetylation, methylation and ubiquitination, are able to improve or inhibit gene transcription, and serve as important factors that modulate immune responses during the immunopathogenesis of inflammatory diseases (Lin et al., 2022).

### Histone acetylation

H3K27 acetylation is considered to be positively correlated with the increased transcriptional activity at gene promoter regions and enhancer elements (Shvedunova and Akhtar, 2022). A whole-transcriptome profiling has indicated the elevation of inflammatory, metabolic, adhesion and oncogenesis pathways in UC-derived compared to non-IBD-derived epithelial organoids, demonstrating increased expression and H3K27 acetylation (H3K27ac) of *LYZ*, *S100P* and *NPSR1* in both UC and colitis-associated colon cancer (Sarvestani et al., 2018). The expression level of ornithine decarboxylase ODC is elevated in human colonic macrophages from UC, CD, colitis-associated dysplasia and colitis-associated colon cancer (Singh et al., 2018). An increased level of H3K9 acetylation (H3K9ac) was manifest in tumor macrophages from *Odc* ^*Δmye*^ mice, further revealing that macrophage ODC could regulate histone modifications and thus augment epithelial injury-associated colitis and colon cancer by damaging the M1 responses (Singh et al., 2018). Furthermore, the low expression of lysine acetyltransferase KAT2B was found in the inflamed colons from UC and CD compared with healthy counterparts. KAT2B knockdown inhibited the occupancy of KAT2B and thereby H4K5 acetylation (H4K5ac) at *IL-10* promoter regions, resulting in the transcriptional silencing of the anti-inflammatory cytokine IL-10 in the inflamed IBD tissues (Bai et al., 2016).

Histone deacetylases (HDACs) are the important enzyme regulating the level of histone acetylation (Haberland et al., 2009). A markedly lower level of histone H3 acetylation was observed in the epithelium of UC patients compared to those from healthy controls (Li et al., 2020). This study also carried out gene set enrichment analysis (GSEA) and found entinostat (MS-275) to be a promising drug targeting UC, which is a specific inhibitor of HDAC1/3. And the inhibition of vitamin D receptor (VDR) significantly restrained the protective ability of MS-275 through interacting with NF-κB p65 and regulating apoptosis, downstream expression of inflammatory cytokines, as well as epithelial barrier function (Li et al., 2020).

### Histone methylation

A study using the mouse model of DSS-induced colitis revealed that the inhibition of histone H4K20 methyltransferase SETD8 promoted the expression of p62, elevated the production of inflammatory molecules, and thereby affected the progression of IBD in patients (Chen et al., 2021a). It was reported that protein arginine methyltransferase PRMT2-mediated H3R8me2a methylation was accountable for the inhibition of SOCS3, and thus suppressed the ubiquitination and degradation of protein TRAF5 mediated by SOCS3. The elevated expression of TRAF5 and the downstream NF-κB/MAPK activation mediated by TRAF5 subsequently promoted the inflammatory state of colitis (Li et al., 2022). Accordingly, elevated expression of PRMT2 was observed in the inflamed tissues of patients from UC (Krzystek-Korpacka et al., 2020), indicating the positive correlation between PRMT2 expression and clinical disease severity of UC. Lonicerin targets histone methyltransferase EZH2 which mediates the decrease of the gene repressive mark H3K27 tri-methylation (H3K27me3) on the promoter of *Atg5*, facilitates the expression of ATG5 protein, prevents the activation of NLRP3 inflammasome, and subsequently alleviates DSS-induced UC (Lv et al., 2021). Another study also demonstrated the role of EZH2 in UC. The inhibition of CCCTC binding factor CTCF prevented the progression of UC by repressing H3K27me3 modification via the deficiency of EZH2 in the *IGFBP5* promoter (Gu et al., 2023). A recent study has found that JMJD3 improves the immune dysfunction of intestinal mucosa by influencing the demethylation of histone H3K27me3, which inhibits the production of inflammatory molecules and thus regulates the differentiation of Th17/Treg cells for the alleviation of inflammation in the mice with experimentally DSS-induced acute UC (Leng et al., 2022).

### Other kinds of histone modification

**FIGURE 2:**
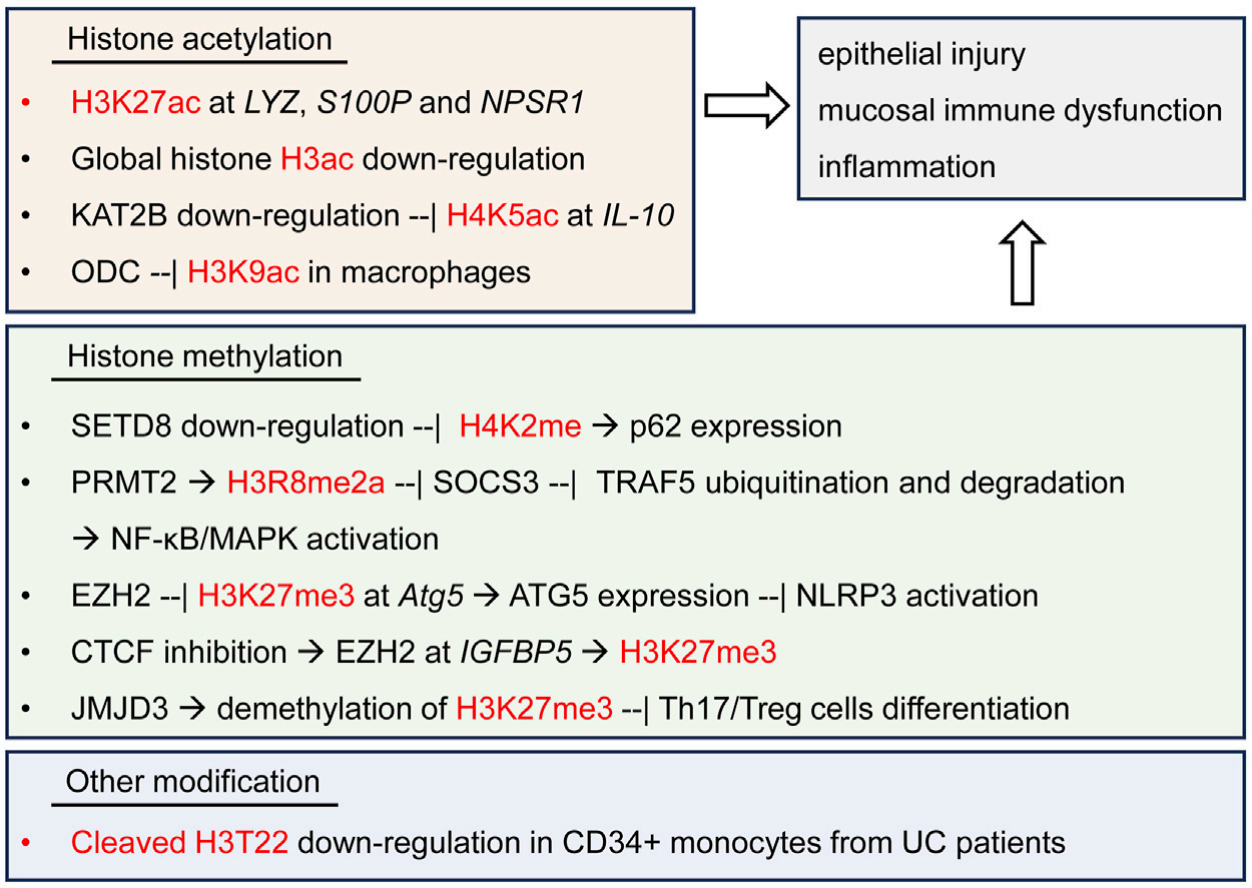
Histone modifications and regulatory signaling pathways in UC. Histone K5, K9, K27 acetylation, K2, K27, R8 methylation and cleaved H3 Thr22 are observed to regulate the expression of multiple inflammatory genes and the signaling pathways in UC patients or DSS-induced experimental UC model, thereby resulting in the injury of colonic epithelium and immune dysfunction.

A current study performed epigenetic profiling by time-of-flight (EpiTOF) of the mononuclear cells in the peripheral blood from 38 patients of UC, 45 patients of CD and 11 healthy controls, and revealed massive heterogeneities in comprehensive histone modifications across multiple types of immune cells (Bai et al., 2023). The increase of several kinds of histone acetylation was observed in a subtype of CD56-bright natural killer (NK) cells from IBD patients. The deficiency of cleaved H3T22 was observed in CD34^+^ monocytes from IBD patients, implying their function to be epigenetically primed for macrophage differentiation (Bai et al., 2023). This study opens a new direction to better exploring the characteristics and epigenetic heterogeneity of global histone modifications in the pathology of UC, by using more high-resolution epigenomic tools, outweighing the traditional ChIP-seq methods (Figure 2).

### MicroRNAs

Non-coding RNAs (ncRNAs) play regulatory roles in shaping cellular activity and multiple cellular processes (Slack and Chinnaiyan, 2019). MicroRNAs (miRNAs) are non-coding single-stranded small RNAs approximately 17–25 nucleotides in length, which serve as key mediators of gene expression in multiple diseases including UC (Schaefer et al., 2015). miRNAs regulate UC mainly through shaping the function of immune cells, affecting the IEC barrier, and regulating the homeostasis between the host and gut microbiota.

miR-141–3p is remarkably upregulated in the IECs of the mice model of UC and might affect the infiltration of inflammatory cells by targeting the chemokines CXCL9 or CXCL16 (Lee et al., 2015). It was also reported that, cycloastragenol suppressed the expression of SphK, MIP-1α, NF-κB, TNF-α, BAX, and cleaved caspase-3 in the UC rat model, associated with the overexpression of miR-143 and BCL2 (Bagalagel et al., 2022). Consequently, cycloastragenol could protect against UC by regulating SphK/MIP-1α/miR-143 axis, hence suppressing the inflammation and apoptosis-related signals (Bagalagel et al., 2022). miR-24 has been reported to be specifically upregulated in the colonic biopsies from patients with active UC, down-regulating the mRNA and protein expression of the tight junction–associated protein Cingulin and thus impairing the intestinal epithelial barrier formation (Soroosh et al., 2019). miR-223 is increased in patients with active UC and may act as a promising signature reflecting diseasse severity (Neudecker et al., 2017). However, another study has reported that myeloid-derived miR-223 inhibits the release of IL-1β by preventing the activation of NLRP3 to alleviate experimental colitis (Zhou et al., 2015), suggesting that miR-223 may have complex functions in UC. miR-31 was reported to be elevated in the colon samples from patients with UC and CD, reducing the inflammatory responses in the epithelial cells by repressing the expression of inflammatory cytokine receptors Il7R and Il17RA, and also the signaling protein GP130 (Tian et al., 2019). miR-182-5p was reported to be a microRNA associated with the progression of colon cancer (Yu et al., 2020), and the inhibition of miR-182-5p could protect against UC by inhibiting HuR-mediated autophagy (Xu et al., 2022b). Another study has reported that high expression of miR-182-5p and SMARCA5 as well as low expression of DNA methyltransferase DNMT3A are tested in patients with UC (Xu et al., 2022c). Specifically, miR-182-5p could repress the Wnt/β-catenin signaling pathway via the methylation of SMARCA5 mediated by DNMT3A, thereby exacerbating UC (Xu et al., 2022c). miR-222-3p was reported to be elevated in the colons from patients with UC and CRC, and the inhibition of miR-222-3p in IECs alleviated the oxidative impairment by targeting BRG1 to activate Nrf2/HO-1 signaling (Wang et al., 2023). miR-214 was proved to be associated with the development of UC and could promote the inflammation, whose deficiency improved the severity of colitis (Liu et al., 2019). And STAT3 was identified as the transcription factor of miR-214 (Liu et al., 2019). A sequential study revealed that ginsenoside Rh2 may function as a kind of treatment of UC by downregulating the level of STAT3/miR-214 (Chen et al., 2021b).

The miR-378a-3p was reported to locate at the first intron of *PPARGC1B* and was differentially regulated in the colonic mucosa of patients with UC (Wu et al., 2008). miR-375-3p was downregulated in the colonic mucosa of patients with UC and CD, and miR-375-3p-mediated upregulation of TLR4 could activate NF-κB signaling and thereby elevate the production of pro-inflammatory molecules (Wu et al., 2019). miR-199a-5p showed significant upregulation in the blood samples from UC patients compared with that of healthy counterparts (Paraskevi et al., 2012). The decreased expression of miR-195-5p could increase the expression of p65, SMAD7, and AP-1, probably explaining the steroid resistance mechanisms in a part of patients with UC (Chen et al., 2015). JAK/STAT signaling pathway was found to be associated to the signaling transduction of plenty of cytokines during the development and immunopathogenesis of UC, and miR-124 could modulate the expression of STAT3 in the colonic samples of children with active UC (Li et al., 2010; Koukos et al., 2013).

A recent study has depicted the differential expressed miRNAs and mRNAs in the colonic mucosa of UC patients through bioinformatic analyses. They further validated that miR-200a, miR-141, and their target genes, including *IRS1*, *HGF*, *SELE*, and *PLEK*, were differentially expressed in UC, identifying these genes as potential biomarkers for targeting UC (Wang et al., 2021). The high-fat diet in the mouse model could promote the release of exosomes with multiple pro-inflammatory factors, including miR-155, from visceral adipose into the intestine to facilitate macrophage M1 polarization and thus exacerbate colitis (Wei et al., 2020). Furthermore, the circRNAs–miRNAs network also plays an essential role in the regulation of UC. hsa_circ_0001021 was reduced and significantly associated with disease severity in UC patients. hsa_circ_0001021 could protect against UC, which influenced the IEC barrier functions by sponging miR-224-5p to upregulate SMAD4 expression (Li et al., 2021). Apart from those miRNAs mentioned above, several miRNAs were also reported to be differentially expressed: miR-16-5p, miR-19a-3p, miR-21-5p, miR-28-5p, miR-29a-3p, miR-30e-5p, miR-31-5p, miR-126-3p, miR-146a-5p, miR-151a-5p, miR-155-5p, and miR-362-3p were upregulated, while miR-192-5p was downregulated in biopsies or blood samples of UC compared to those of healthy counterparts (Takagi et al., 2010; Schaefer et al., 2015; Yarani et al., 2022).

### LncRNAs

Long non-coding RNAs (lncRNAs) are known as transcripts longer than 200 nucleotides without protein-coding capacity (Statello et al., 2021). LncRNAs are able to bind and interact with DNA, RNA or protein, and epigenetically regulate gene expression majorly at the transcriptional and post-transcriptional levels, through the regulation of transcription, mRNA stability, protein translation and subcellular location, which have been reported to play essential roles in different biological processes involved in immune responses and disease development (Statello et al., 2021).

LncRNA *CDKN2B-AS1* is commonly decreased in patients with UC. Forming linear and circularized RNA transcripts, the downregulation of *CDKN2B-AS1* causes elevated barrier integrity in the colon by inhibiting the expression of Claudin-2 (Rankin et al., 2019).

lncRNA *H19* and miR-675-5p was upregulated while VDR was downregulated in samples of UC patients compared to those of healthy counterparts. The overexpression of *H19* inhibited the expression of tight junction proteins and VDR *in vitro*, mediated by miR-675-5p targeting the 3′UTR of *VDR* mRNA (Chen et al., 2016). A previous study has examined the differential expression of lncRNAs in UC patients, and identified that the lncRNA *IFNG-AS1* was related to the susceptibility loci SNP rs7134599 of IBD, and could positively regulate the expression of inflammatory cytokine IFNG by CD4^+^ T cells (Padua et al., 2016). By detecting the mRNA expression of various lncRNAs in the tissues and plasma samples from patients with UC and CD, a study found that *LINC01272* and *KIF9-AS1* were considerably upregulated in tissues and plasma samples from IBD patients compared to those from healthy counterparts, whereas the expression of *DIO3OS* were markedly downregulated (Wang et al., 2018). This study suggested that *LINC01272*, *KIF9-AS1* and *DIO3OS* might serve as promising diagnostic signatures for UC and CD. Another study identified 329 upregulated and 126 downregulated lncRNAs in the tissues of active UC compared with those of healthy controls, among which *BC012900*, *AK001903*, and *AK023330* were the most significantly upregulated and *BC029135*, *CDKN2B-AS1*, and *BC062296* were the most downregulated transcripts (Wu et al., 2016). Besides, the overexpression of *BC012900* could repress proliferation and facilitate apoptosis in the epithelium (Wu et al., 2016), implying its role in elevating intestinal inflammation. *Lnc-UC* is a colitis-associated cycling lnRNA uncovered in mice and humans (Wang et al., 2020). It is induced by NF-κB signaling pathway in the colitis, which can increase the transcription of *Rev-erbα* for the alteration of circadian gene expression. *Lnc-UC* physically interacts with Cbx1 protein to inhibit its function in gene silencing via H3K9me3, thus promoting the transcription of *Rev-erbα* and linking circadian clock to colitis (Wang et al., 2020).

A study has demonstrated that multiple immune-associated pathways were implicated in UC, especially those involved in innate immunity, via GSEA of samples from patients with active UC and heathy counterparts (Xu et al., 2022d). Comprehensive bioinformatics analysis and *in vivo* validation using the mouse model of colitis identified *IL1B*, *CXCL1*, *MMP1* and *MMP10* as markers of UC. Moreover, they predicted a competing endogenous RNA (ceRNA) network of the lncRNA *XIST*-miR-9-5p/miR-129-5p/miR-340-5p-NF-κB axis to regulate the expression of NF-κB (Xu et al., 2022d), thus offering new insights for the combinational therapies of UC. *Lnc-ITSN1-2* could serve as a ceRNA to sponge miR-125a, thus promoting the expression of IL-23R, promoting CD4^+^ T cell responses and increasing the inflammatory cytokine profiles of UC (Nie and Zhao, 2020). A recent study uncovered UC-related lncRNAs, including *ZFAS1*, *MIR4435-2HG*, *Pvt1*, and *IL6-AS1*, probably regulated by upstream differentially methylated regions (DMRs). And they further identified genes implicated in the inflammatory immune responses downstream of DMR-modulated lncRNAs, including *CCL18*, *SERPINB1*, and *SLC15A4* (Fenton et al., 2023). Therefore, the interplay between the expression of lncRNAs in UC and the regulation of DNA methylation may enhance our knowledge of the immunopathogenesis in UC.

### Epigenetics-associated therapeutic strategies of UC

There have been various novel therapeutic approaches and drugs for the manipulation and treatment of UC, such as JAK inhibitors (tofacitinib, upadacitinib, filgotinib, TD-1473), phosphodiesterase inhibitors (apremilast), sphingosine receptor modulators (fingolimod, ozanimod and etrasimod), anti-adhesion molecules (natalizumab, vedolizumab, etrolizumab and ontamalimab) and anti-interleukin antibodies (ustekinumab targeting IL-12 and IL-23, mirikizumab targeting IL-23) (Hirten and Sands, 2021). Besides, as an important transcription factor responsible for the control of cellular defense, Nrf2 regulates the progression of UC through multiple mechanisms, whose expression is also regulated by epigenetic modifications (Peng et al., 2023). There have been many natural compounds and synthetic chemicals regulating the function of Nrf2 on UC, and novel drugs need to be developed to potentiate the defensive effects of Nrf2 (Peng et al., 2023).

miRNAs that play essential roles in the regulation of signaling transduction pathways during the development of UC have been the promising therapeutic targets. Obefazimond (ABX464) has been originally used as an orally-administered small molecule drug anti-human immunodeficiency virus (HIV) infection (Campos et al., 2015). Recently, it seemed to be safe and well-tolerated in phase II placebo-controlled induction trials to treat patients with moderate-to-severe UC (Vermeire et al., 2021; Vermeire et al., 2022; Vermeire et al., 2023), showing a great promise in the therapeutic application. Obefazimond forms the interaction with the cap binding complex, allowing for the enhanced selective splicing of miR-124 (an anti-inflammatory microRNA mentioned above), resulting in a cascade downregulation of pro-inflammatory cytokines and chemokines (Vermeire et al., 2021; Vermeire et al., 2023).

**FIGURE 3:**
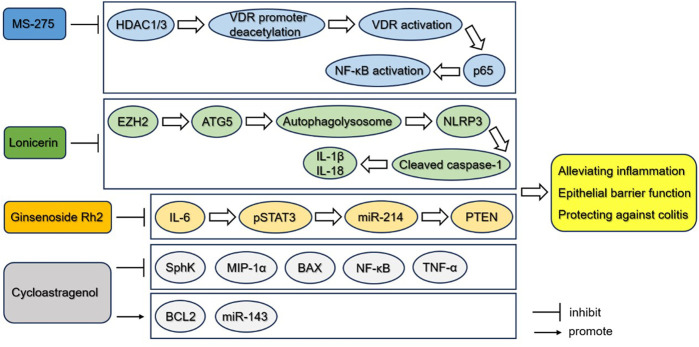
Small molecules potentially targeting epigenetic pathways for the treatment of UC. MS-275 inhibits the deacetylation of VDR promoter and thereby activates VDR, which directly interacts with p65 and affects downstream expression of NF-κB-related inflammatory cytokines, cellular apoptosis and epithelial barrier function. Lonicerin can be considered as an anti-inflammatory epigenetic agent that targets histone methyltransferase EZH2 mediating the decrease of the gene repressive mark H3K27me3 on the promoter of *Atg5* and inducing the expression of ATG5, and inhibits the activation of NLRP3 inflammasome. Ginsenoside Rh2 may function as a kind of treatment of UC by inhibiting the activation of STAT3/miR-214/PTEN. Cycloastragenol treatment upregulates the expression of SphK, MIP-1α, BAX, NK-κB and TNF-α, while downregulates the expression of BCL2 and miR-143. These small molecules may serve as potential agents alleviating inflammation and protecting against colitis in UC.

As was mentioned above, many kinds of small molecules could be promising drugs for therapeutic applications of UC, such as ginsenoside Rh2 as an STAT3 inhibitor, MS-275 as an HDAC inhibitor, lonicerin as an inhibitor of histone methyltransferase EZH2, and cycloastragenol as an anti-inflammatory molecule, whose effect have been validated in mice or rat models. Efforts should be made to develop novel treatment modalities and approaches, so that the clinical applications of these small molecules regulating epigenetic events should be further evaluated. Besides, the identification of novel biomarkers based on epigenetic studies will help monitor disease activity and ultimately lead to advances in the treatment of patients with UC (Figure 3).

### Future perspectives

It has been widely studied about the role of miRNAs as diagnostic and therapeutic targets recently. miRNA-based therapeutic strategies have offered a novel perspective for the futural intervention of patients suffered from IBD (Aggeletopoulou et al., 2023). However, there are many challenges in the development and clinical application of miRNA-based drugs. Specifically, the high specificity, efficiency, off-target side effect, and safety when delivered to the inflamed regions remain major concerns, which can collectively influence many downstream genes and relevant signaling pathways. Accurate and efficient delivery of the specific miRNAs to the target cells or tissue regions will contribute to eliminate the side effects and promote therapeutic effects *in vivo*. Therefore, the delivery strategy for miRNAs is also needed to be concerned.

The discovery of the cytosine derivatives, i.e., 5hmC, 5fC and 5caC, strongly expands our knowledge related to the modulation of gene expression. The specific roles they might play in the regulation of gene expression need to be further investigated. Due to the fact that epigenetic modifications are reversible, we can potentially reverse the relevant disease processes by targeting epigenetic events to develop therapeutic approaches in the future. Moreover, the interrelationship of different kinds of epigenetic regulation should be explored to better study the effect of epigenetics on the immunopathogenesis of UC.

Despite of the regulatory mechanisms we summarized in the current review, there are still additional aspects that should be considered respecting the instability of epigenetic mechanisms. Attention should be paid to the differential epigenetics potentially caused by the specific factors, such as ethnicity, age, sex, lifestyle, comorbidity, environment and treatment. The impact caused by these factors is gaining importance in terms of their influence on the epigenetic signatures, the outcome, disease progression, and response to UC treatments, which should therefore be considered when designing relevant studies and developing therapeutic strategies. The development of novel, cutting-edge research tools and methods for epigenetic studies allows for the possibility to identify specific patterns of DNA and histone modifications, chromatin alterations, as well as non-coding RNAs with clinical value in the treatment of UC.
